# AGE DIFFERENCES IN EMOTION UTILITY KNOWLEDGE: COMPARING DISCRETE VERSUS DIMENSIONAL MODELS

**DOI:** 10.1093/geroni/igad104.2157

**Published:** 2023-12-21

**Authors:** Nikolina Kravljaca, Jennifer Stanley, Eric Allard

**Affiliations:** The University of Akron, Barberton, Ohio, United States; University of Akron, Akron, Ohio, United States; Cleveland State University, Cleveland, Ohio, United States

## Abstract

Do young and older adults have the same metacognitive schema for emotional utility? We examined young and older adults’ knowledge of which emotion would be best for achieving a goal (e.g., anger for confrontation). We captured responses using both a discrete emotions framework (e.g., happy; Ekman 1992) and a dimensional/circumplex model approach (e.g., arousal and valence; Russell, 1980) to determine which framework is most useful for understanding age differences in utilitarian emotion knowledge. Seventy-five young and 66 older adults indicated the most useful emotion when playing video games associated with different goals (Approach, Avoid, Confront, Analyze). Participants were asked to rate discrete emotions as well as identify the best combination of preferred valence and arousal on an affect grid. Two separate mixed-design ANOVAs were conducted for discrete emotion and dimensional ratings. Young and older adults exhibited similar preferences for both the discrete and circumplex model and chose the emotion with the most expected utility for each goal (e.g., happy for approach). One interesting age difference emerged where young adults endorsed the expected utilitarian emotion of sadness for the analyze goal to a greater extent than older adults. For the affect grid ratings, age differences were driven by arousal but not valence. We discuss how a discrete emotions approach may allow some clarity and a common language for interrogating the role of aging in utilitarian emotionality but may lack the nuance provided by the circumplex model.

